# Resident Versus Attending Prenatal Care Models: an Analysis of the Effects of Race and Insurance on Appointment Attendance

**DOI:** 10.1007/s40615-023-01665-8

**Published:** 2023-06-12

**Authors:** Veronica Fitzpatrick, Anne Rivelli, Iridian Guzman, Kim Erwin

**Affiliations:** 1 Advocate Aurora Research Institute, 3075 Highland Parkway, Downers Grove, IL 60515 USA; 2 Advocate Aurora Health, Downers Grove, IL USA; 3 Illinois Institute of Technology Institute of Design, Chicago, IL USA

**Keywords:** Prenatal care, Pregnancy, Healthcare utilization, Adherence, Healthcare disparities, Race

## Abstract

**Objective:**

To describe patient differences by prenatal care (PNC) model and identify factors that interact with race to predict more attended prenatal appointments, a key component of PNC adherence.

**Methods:**

This retrospective cohort study used administrative data targeting prenatal patient utilization from two OB clinics with different care models (resident vs. attending OB) from within one large midwestern healthcare system. All appointment data among patients receiving prenatal care at either clinic between September 2, 2020, and December 31, 2021, were extracted. Multivariable linear regression was performed to identify predictors of attended appointments within the resident clinic, as moderated by race (Black vs. White).

**Results:**

A total of 1034 prenatal patients were included: 653 (63%) served by the resident clinic (appointments = 7822) and 381 (38%) by the attending clinic (appointments = 4627). Patients were significantly different across insurance, race/ethnicity, partner status, and age between clinics (*p* < 0.0001). Despite prenatal patients at both clinics being scheduled for approximately the same number of appointments, resident clinic patients attended 1.13 (0.51, 1.74) fewer appointments (*p* = 0.0004). The number of attended appointments was predicted by insurance in crude analysis (*β* = 2.14, *p* < 0.0001), with effect modification by race (Black vs. White) in final fitted analysis. Black patients with public insurance attended 2.04 *fewer* appointments than White patients with public insurance (7.60 vs. 9.64) and Black non-Hispanic patients with private insurance attended 1.65 *more* appointments than White non-Hispanic or Latino patients with private insurance (7.21 vs. 5.56).

**Conclusion:**

Our study highlights the potential reality that the resident care model, with more care delivery challenges, may be *underserving* patients who are inherently more vulnerable to PNC non-adherence at care onset. Our findings show that patients attend more appointments at the resident clinic if publicly insured, but less so if they are Black than White.

## Introduction

Non-adherence to prenatal care (PNC) is a well-documented preventable cause of patient morbidity and mortality and a source of wasted healthcare resources [1–4]. This is likely due to the shift of routine PNC in the United States (US) to a practice-level service that relies on well-established prenatal guidelines to promote patient PNC plan adherence, most broadly defined as “following the healthcare utilization recommendations of their OB providers,” which in obstetrics (OB) is based on completion of OB provider visits at least once in the first trimester, twice in the second trimester, and five times throughout the third trimester (≥ 8 visits total) for *minimal* adherence [1, 2, 5, 6]. PNC plan adherence is intrinsically linked with maternal health outcomes, of which the US has the highest maternal mortality rate among developed countries, with rates that have been steadily increasing for 20 years [7]. PNC is indispensable for assuring healthy pregnancy outcomes; however, not all prenatal patients utilize and experience PNC similarly [2, 8, 9].

Socioeconomic status has been identified as a significant contributor to the racial/ethnic disparities in OB. Studies that have attempted to understand the complex and layered relationship between race/ethnicity, socioeconomic status, and prenatal care outcomes have demonstrated that racial disparities may be context dependent. Although higher SES can have a protective effect on maternal and infant outcomes, whether it eliminates racial disparities seen in outcomes depends on the care context [10, 11]. One study looking at the interaction effects of race, SES, and neighborhood on infant birthweight found that the gap in lower birthweight between infants born to Black and White women was moderately reduced when both were of similar SES and living in racially concordant neighborhoods; on the contrary, higher SES Black women living in disproportionately White neighborhoods had the worst outcomes in infant birthweight [11]. While SES and race/ethnicity cannot account for outcomes alone, the intersection between socioeconomic status and race should be further explored, particularly in different care contexts. Identifying and understanding the complex web of factors that result in disparate prenatal and maternal outcomes is critical, particularly between Black and White women, especially given that as recent as 2020, US maternal mortality rate for Black women was 2.9 times greater than that for White women [12].

It has long been suspected and recently documented that racism, including structural racism, is a likely contributor to the widening disparity in maternal health outcomes between White and Black women [7, 10–14]. One way in which structural racism is observed in healthcare is through the overrepresentation of residency clinics in predominantly underserved communities made up of non-White racial or ethnic groups minorities [13, 14]. As facilities where physician trainees are learning to provide clinical care, residency clinics are known to face various challenges for patients, such as having long wait times [15] and inadequate resources [14, 16]. These realities also have a significant impact on residents’ ability to appropriately care for their patients. Furthermore, residency clinics also face greater challenges in providing continuity of care since residents are often shuffled between different clinics and generally given limited time to establish trusting relationships with their patients [14, 16]. The challenges that residency clinics face cannot only impact care quality and associated OB outcomes, but these challenges can also affect the quantity of appointments attended by patients, particularly among those already struggling to adhere due to barriers defined by SES and resources.

The following study explores data extracted from a larger cohort study [17] to describe differences in patient characteristics and appointment patterns by PNC model and to identify factors that interact with race to predict more attended prenatal appointments, a key component of treatment adherence to PNC plans.

## Methods

For this study, we explored two populous Chicago-based OB clinics within a large Midwest healthcare system, each with different care models—one operates using resident OBs as the primary providers (“Resident clinic”) and the other operates with attending OBs as the primary providers (“Attending clinic”). Generally, the resident clinic serves prenatal patients who are publicly insured, predominantly Hispanic, high and low risk with about 4 resident OBs on rotation as staff at any given time. The attending clinic serves prenatal patients who are privately insured, predominantly White, high and low risk, with about four attending OB providers on staff. Administrative data of prenatal patient utilization occurring between September 2, 2020, and December 31, 2021, at the included clinics were extracted from the healthcare system’s electronic medical records (EMR) to coincide with the larger cohort study of which this data was derived [17]. Prenatal patients were identified from pregnancy-specific ICD-10 codes, and all encounters (i.e., appointments) scheduled and attended at each of the two involved clinics within the timeframe were included in the dataset. There were no exclusion criteria. While patients were technically free to choose the clinic they visited, clinic choices were limited by patient insurance, which is typical in healthcare. Study approval was granted by the health system’s institution review board (#20-264E) and funded through an intramural grant (#GR-00000091).

### Data

Patient characteristics were collected on all prenatal patients, including the following: age: continuous; race: White, Black, or Asian; ethnicity: Hispanic or Latino or non-Hispanic or Latino; insurance status: public or private; and marital status: single, significant other, divorced, legally separated, widowed, or married/civil union. Appointment data for all patients were captured, including the following: appointments (dates): appointment check-in and check-out (dates and times); cancelation indicator: yes or no; and reasons for canceled appointments. Per standard reporting practices, race and ethnicity were combined, creating four race/ethnicity categories: White non-Hispanic or Latino (“White”), Black non-Hispanic or Latino (“Black”), Asian non-Hispanic or Latino (“Asian”), and Hispanic or Latino. Marital status was collapsed as partner status: partner or no partner; and race/ethnicity and partner status were used in analyses. Furthermore, appointment check-in and check-out dates and times were used to calculate appointment length, or total time spent in clinic, as a continuous variable (minutes). Given research indicating inequity in PNC adherence and wait times [9, 15], this study aimed to identify differences in PNC utilization, specifically appointment attendance and length, by care model and to explore contributors that interact with race/ethnicity to help explain PNC attendance.

### Statistical Analyses

Analyses were performed using SAS version 9.4 (SAS Institute, Cary, NC). Patient characteristics and appointment cancelations are described as counts (percentages) among the entire sample and by care model, with Pearson chi-square analyses used to generate *p*-values to reflect statistically significant differences in patient characteristics by care model and logistic regressions used to generate odds ratios. Patient appointments are described as means (standard deviations) and medians (interquartile ranges), with Student’s *t*-tests generated to calculate mean differences and Student’s *t*-tests or Mann–Whitney *U* (also known as Wilcoxon rank sum) tests used to generate *p*-values reflecting statistically significant differences. Multivariable linear regression was performed to identify predictors of attended appointments. All patient characteristic variables (race/ethnicity, insurance, partner status, age) were explored as potential predictors, with effect modification of insurance on race/ethnicity pursued given research suggesting that racial disparities are often socioeconomic context dependent. Indicator variables were created as necessary. Alpha of *p* < 0.05 was considered statistically significant for all analyses.

## Results

### Demographics

The sample included 1034 prenatal patients who were scheduled for at least one prenatal appointment at either the resident clinic (*N* = 653) or attending clinic (*N* = 381). Overall, the sample was prenatal patients who were primarily Hispanic or Latino (47.49%), married or in a civil union (56.09%) with private insurance (53.68%), and a mean age of 31.07 (5.77). Among 653 (63.15%) patients served at the resident clinic, prenatal patients were primarily Hispanic or Latino (53.45%), either married or in a civil union (45.79%) or single (45.33%) with public insurance (67.53%), and a mean age of 30.26 (6.03). Among the 381 (36.85%) patients served at the attending clinic, prenatal patients were primarily White (51.44%), married or in a civil union (73.75%) with private insurance (90.03%), and a mean age of 32.46 (5.01) (see Table 1).

**Table 1:** Demographics of sample, overall, and by care model at care onset (encounter 1)

Variables	Care model clinic^Ϯ^		Odds ratio^#^	*p*-value^+^
	Resident (*N* = 653, 63.15%)	Attending (*N* = 381, 36.85%)		
*Race/ethnicity*				
Hispanic or Latino	349 (53.45%)	142 (37.27%)	3.60 (2.68, 4.82); p = 0.132	< 0.001**
Black, NH	135 (20.67%)	18 (4.72%)	10.97 (6.40, 18.80); p < 0.001	
Asian, NH	35 (5.36%)	25 (6.56%)	2.05 (1.17, 3.58); p = 0.069	
White, NH	134 (20.52%)	196 (51.44%)	REF	
*Insurance*				
Public	441 (67.53%)	38 (9.97%)	18.78 (12.93, 27.26)	< 0.001**
Private	212 (32.47%)	343 (90.03%)	REF	
*Partnership status*				
No partner	303 (46.40%)	82 (21.52%)	3.16 (2.37, 4.21)	< 0.001**
Partner	350 (53.60%)	299 (78.48%)	REF	
*Age (mean (SD); median (IQR))*	30.26 (6.03); 31 (26–35)	32.46 (5.01); 33 (30–36)	− 2.21 (− 2.88, − 1.52)	< 0.001**

^Ϯ^Describes column percentages to better reflect differences by care model

^**^Statistically significant at *p* < 0.001

^#^ORs generated from logistic regressions

+ *P*-values generated from chi-square tests or ^Fisher’s exact tests if cell count < 5

Differences by care model revealed that, relative to patients served in the attending clinic, patients served in the resident clinic had 10.97 (6.40, 18.80) times greater odds of being Black relative to White, 18.78 (12.93, 27.26) times greater odds of having public insurance, and 3.16 (2.37, 4.21) times greater odds of having no partner and were, on average, 2.21 (1.52, 2.88) years younger. All these differences in patient characteristics by care model were statistically significant at *p* < 0.001 (see Table 1 for more details).

### Appointments Scheduled

Among the entire sample, there were 12,449 total appointments scheduled among all 1034 unique patients, with 7822 appointments among the 653 unique patients served in the resident clinic and 4627 appointments among the 381 unique patients served in the attending clinic. Among the entire sample, there was an average of 12.04 (7.98) appointments *scheduled*. The average number of scheduled appointments was 11.81 (8.58) in the resident clinic and 12.43 (6.84) in the attending clinic. There was no statistical difference in overall scheduled appointments between clinics, with no statistical difference in number of scheduled appointments between clinics.

### Appointments Attended

Among the entire sample, there was an average of 7.99 (5.08) appointments *attended*. The average number attended appointments was 7.58 (5.28) in the resident clinic and 8.70 (4.61) in the attending clinic. Patients served in the resident clinic attended 1.13 (0.51, 1.74) fewer appointments relative to patients served in the attending clinic (*p* < 0.001). The median appointment length was 86 (63) minutes, with 87 (60) median minutes among patients served in the resident clinic and 37 (29) median minutes among patients served in the attending clinic. While median appointment length minutes were statistically different by care model at *p* < 0.0001, it is important to note that suspected differences in data collection processes by clinic resulted in significant missing data in the attending clinic data; however, given the finding, it is important to present but was not used in any of our multivariate analyses. Furthermore, the disparity in data collected on appointment length either represents a true difference, which is believed based on observation, and/or the clinics collect and document data differently, which could inhibit detection of administrative comparisons of some variables between clinics. Findings are reported in Table 2 to document differences by clinic given potential implications surrounding clinic practices by care model.

**Table 2:** Mean appointments by care model

Variables	Resident (*N* = 653, encounters = 7822)	Attending (*N* = 381, encounters = 4627)	Mean Diff	*p*-value^+^
*Appointments (scheduled)*	11.81 (8.58); 11 (4–17)	12.43 (6.84); 13 (7–17)	− 0.63 (− 1.58, 0.32)	0.1936
	Resident (encounters = 4991)	Attending (encounters = 3228)	Mean Diff	*p*-value
*Appointments (attended)*	7.58 (5.28); 7 (3–12)	8.70 (4.61); 9 (5–12)	− 1.13 (− 1.74, − 0.51)	< 0.001**
	Resident (encounters = 4823)	Attending (encounters = 61)	Mean Diff	*p*-value
*Appointment length (mins)*	177.79 (415.42); 87 (62–124)	37.74 (16.90); 37 (23–52)	140.10 (127.60, 152.50)	< 0.001**

^**^Statistically significant at *p* < 0.0001

+ *P*-value generated from Mann–Whitney *U*, or Wilcoxon rank sum, test

### Appointments Canceled

**Fig. 1:**
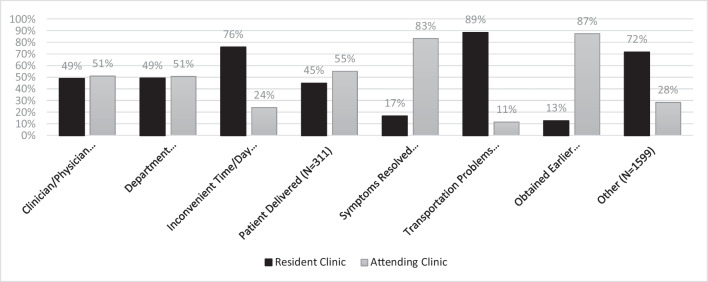
Cancelation reasons by care model (*N* = 3807 total encounters)

Across all 12,449 appointments, 3807 (30.58%) were canceled among this sample, with 1.12 (1.03, 1.21) times greater odds of appointments being canceled at the resident clinic relative to the attending clinic (*p* = 0.005). Furthermore, among all 3807 canceled appointments, 16.52% of them were canceled due to patients not showing up and accordingly canceled; odds of no-show appointments were 3.23 (2.66, 4.16) times greater at the resident clinic (*p* < 0.001). Among the reported reasons for cancelation, “inconvenient times/day for patient” was recorded most frequently (1005 appointments; 26.40% overall), with 76.02% occurring at the resident clinic compared to 23.98% at the attending clinic. Furthermore, it is interesting to note that among canceled appointments with “transportation problems” recorded (70 appointments; 1.84% overall), 88.57% occurred at the resident clinic (see Table 3 and Fig. 1 for more details).

**Table 3:** Cancelations by care model

Cancelations by care model	Resident (*N* = 653, encounters = 7822)	Attending (*N* = 381, encounters = 4627)	Odds ratio	*p*-value
*Cancelations*	2461 (64.64%)	1346 (35.36%)	1.12 (1.03, 1.21)	0.005*
*Missed/no-show appointment*	527 (83.78%)	102 (16.22%)	3.23 (2.66, 4.16)	< 0.001**
*On-time cancelation*	1934 (60.86%)	1244 (39.14%)	0.30 (0.24, 0.38)	< 0.001**

^**^Statistically significant at *p* < 0.0001

^*^Statistically significant at *p* < 0.05

### Resident Clinic Only Results

**Fig. 2:**
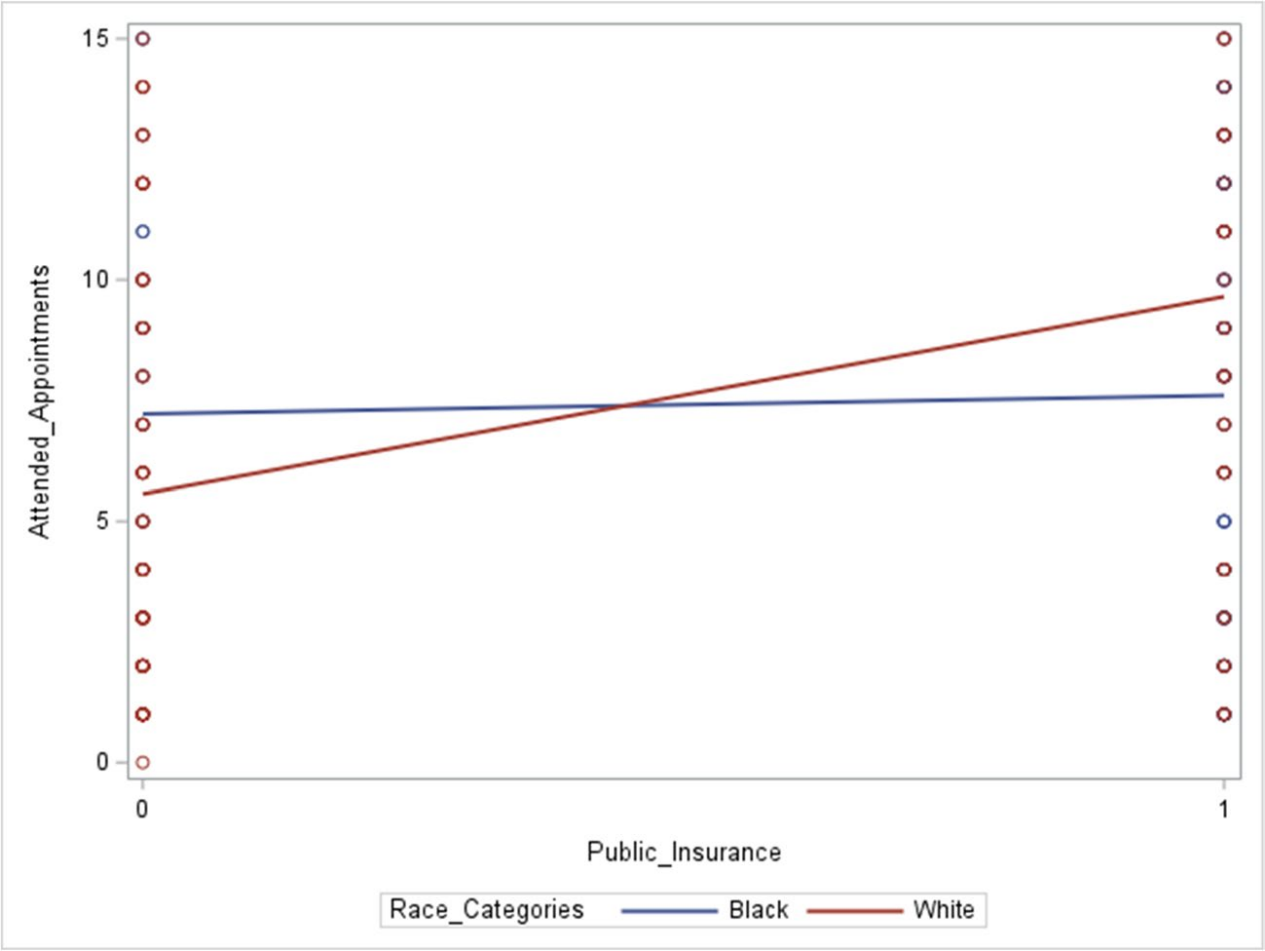
Effective modification of insurance on attended appointments by race

Despite prenatal patients at the resident and attending clinics being scheduled for approximately the same number of appointments, there were significant differences in the mean number of *attended* appointments between these clinics, with patients at the resident clinic attending fewer appointments overall. Given fewer attended appointments and more diversity in patient characteristic factors, including race/ethnicity, insurance, and partnership status, we decided to explore factors that interact with race to predict poor PNC attendance at the resident clinic only; thus, a multivariable linear regression was performed. All patient characteristics were explored in crude and adjusted analyses, and insurance was the single patient characteristic that was significantly associated with attended appointments. Stepwise selection with entry and stay values of *p* < 0.05 supported insurance as the only variable that significantly predicted attended appointments at the resident clinic. Moderation by race/ethnicity, specifically Black vs. White patients, was then explored within the resident clinic model using stratified models and interaction plots, revealing interactions with insurance (Fig. 2).

Subsequently, insurance by race/ethnicity (White vs. Black) interaction variables were entered into the model. The final model included insurance (public vs. private), race/ethnicity (White vs. Black), and insurance by race/ethnicity as significant variables predicting attended appointments at the resident care model clinic. Specifically, the final fitted regression model was the following: Attended appointments = 5.56 + 4.09 (Public insurance) + 1.65 (Black) +  − 3.70 (Public insurance*Black). The final model was statistically significant (adjusted *R*^2^ = 0.0572, *F*(3, 265) = 6.42, *p* < 0.001). The model revealed that having public insurance significantly predicted a greater number of attended appointments (*β* = 4.09, *p* < 0.001) and that the interaction between insurance and race (Black vs. White) significantly predicted attended appointments (*β* =  − 3.70, *p* < 0.001) (see Table 4).

**Table 4:** Linear regression model identifying predictors of attended appointments at resident care model clinic

*Crude models* +	*Intercept (SE)*	*Estimate (SE)*	*95% CI*	*p-value*	*Adj R*^*2*^	*F*	*df*
Public insurance	6.08 (0.36)	2.14 (0.44)	1.29, 3.00	< 0.001**	0.0341	24.04	1, 651
White NH	7.72 (0.23)	− 0.94 (0.51)	− 1.95, 0.07	0.068	0.0036	3.34	1, 651
Black NH	7.53 (0.23)	− 0.02 (0.52)	− 1.03, 0.99	0.963	− 0.0015	0.00	1, 651
Hispanic NH	7.24 (0.30)	0.55 (0.42)	− 0.27, 1.37	0.188	0.0011	1.73	1, 651
Asian NH	7.51 (0.21)	0.41 (0.92)	− 1.41, 2.22	0.660	− 0.0012	0.19	1, 651
No partner	7.45 (0.28)	0.16 (0.42)	− 0.66, 0.98	0.696	0.0002	.0.15	1, 651
Age	8.46 (1.06)	− 0.03 (0.03)	− 0.10, 0.04	0.371	− 0.0003	0.80	1, 651
*Adjusted model*	*Estimate (SE)*	*95% CI*	*p-value*	*Model p-value*	*Adj R*^*2*^	*F*	*df*
Intercept	5.56 (0.53)	4.52, 6.61	< 0.001	< 0.001**	0.0572	6.42	3, 265
Public insurance	4.09 (0.97)	2.18, 5.99	< 0.001				
Black NH^	1.65 (1.05)	− 0.41, 3.72	0.116				
Public insurance*Black NH	− 3.70 (1.42)	− 6.50, − 0.91	0.009				

+ All variables in crude models are binary, with 1 = yes and 0 = no, except Age as continuous

^Black NH in adjusted model represents Black vs. White

^**^Statistically significant at *p* < 0.001

**Fig. 3:**
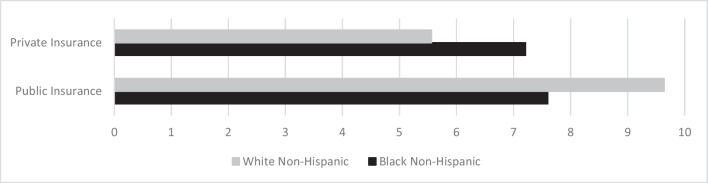
Final model estimates predicting attended appointments by insurance and race/ethnicity at the resident clinic

Patients with public insurance attended, on average, 4.09 more appointments than those with private insurance; however, looking closely at the interaction, while Black patients with public and private insurance attended similar number of appointments overall (7.60 vs. 7.21, respectively), Black patients with public insurance attended 2.04 *fewer* appointments than White patients with public insurance (7.60 vs. 9.64) and Black non-Hispanic or Latino patients with private insurance attended 1.65 *more* appointments than White non-Hispanic or Latino patients with private insurance (7.21 vs. 5.56) (Fig. 3).

## Discussion

Where and what quality of healthcare one receives is dictated by insurance, which is no different in OB care. As stated previously, it is well-documented that, with some exception, privately insured prenatal patients are most likely to receive adequate PNC, given they have access to more flexible options, like being treated at clinics with experienced OB providers. In general, privately insured patients tend to be White with fewer medical issues and more resources, unlike patients who have public insurance and tend to be lower-income, minority, and have existing medical comorbidities and external barriers to adequate care. Plainly put, patients with public insurance are the most socially vulnerable and, due to public insurance, are more often served by the resident care model, despite the inherent reality of the clinical rotations, teaching clinic procedures, and lack of continuity of care signature to this model. This model has the biggest care delivery challenges for both trainees and patients, which, bottom line, has ramifications for the care provided, particularly for patients already at a disadvantage.

This study aimed to describe patient characteristics and appointment patterns across two clinics with vastly different care models. Resident clinic patients were more likely to be Black non-Hispanic or Latino, have public insurance, have no partner, and be younger than patients served at the attending clinic. Furthermore, while both the resident and attending clinics *scheduled* the same number of appointments for their patients (~ 12), consistent with the American Colleges of Obstetricians and Gynecologists (ACOG) recommendations, there were significant differences in *attended* appointments, with prenatal patients at the resident clinic attending fewer scheduled appointments. This is not entirely unexpected given that resident clinics, more broadly, tend to see Medicaid patients in higher proportions than other clinic care model types. Medicaid (i.e., public) insurance, a strong proxy for income status, would then infer more external barriers to appointment attendance, as cited in current research [18, 30]. This study corroborated this research, showing that two of the main reasons for no-shows/cancelations at the resident clinic were due to inconvenient appointment time and transportation issues whereas the attending clinic patients canceled appointments for symptom resolution and more convenient appointment times, speaking to disparity in the patient populations served at these clinics, respectively. Potentially compounding this issue is the indication that prenatal patients tend to spend significantly more time at the resident clinic while attending their appointments, which exasperates transportation and appointment time inconveniences, though our data had some limitations.

Diving deeper into predictors of appointment attendance at the resident clinic, insurance was the only significant predictor of attended appointments at the resident clinic *before* accounting for any interaction with race. One may take away that public insurance helps improve attendance within a resident care model; however, when accounting for this interaction, the impact of insurance (public vs. private) on the number of attended appointments was moderated by race/ethnicity (Black vs. White) at the resident clinic. Comparing Black and White patients at the resident clinic, while both Black and White patients with public insurance attended more appointments than their privately insured counterparts, among those with public insurance, Black patients attended 2.04 fewer appointments than White patients, suggesting increased barriers by race. Conversely, among those with private insurance, Black patients attended 1.64 more appointments than White patients, potentially indicating that White patients go elsewhere for care when given the option while Black patients tend to remain in the clinic they originated care, regardless of lesser care. It is also important to note that Black patients attended similar number of appointments at the resident clinic, regardless of insurance. Taken together, these findings may suggest that Black patients prioritize care convenience over quality regardless of options, reiterating potential increased barriers to care they may face relative to White patients. Most poignantly, our findings show that patients attend more appointments at the Resident clinic if publicly insured, but less so if they are Black than White. On top of known barriers associated with public insurance as a proxy for poorer income/resources, these findings highlight increased barriers by race, more negatively affecting publicly insured Black patients’ OB care attendance and subsequent care delivery compared to publicly insured White patients. Care delivery challenges impact the PNC experience, PNC adherence, and perhaps ultimately maternal and child outcomes [26, 30–33].

### Implications

The most direct way to improve equity in PNC is to have an even distribution of prenatal patients, regardless of race/ethnicity and insurance, into all types of OB care models—resident/teaching, attending OB, and midwife. Another option may be putting prenatal patients into a PNC model based on something entirely external to sociodemographic or other factors known to be associated with inherent barriers to care. Residents should not predominantly see socially vulnerable prenatal patients and, similarly, attending OBs should not predominantly (in some cases only) treat privately insured, White prenatal patients, especially in a community-based healthcare system. Until the most vulnerable prenatal patients have the systemic opportunity to see the optimal provider for their specific set of circumstances, there will continue to be an equity gap in maternal and child health outcomes.

### Limitations

This study collected data on a 16-month time period, which may result in our study patient sample not having their entire PNC journal captured, depending upon when they initiated care within this time period. Furthermore, the study period occurred during the COVID-19 pandemic, which altered clinical operations broadly, but for the clinical sites included in this study. Although COVID-19 disproportionately impacted patients overall, but especially people of color, this study was not able to account for these differences, specifically. Due to data collection discrepancies, there was limited data on appointment length at the attending clinic, but enough to make a preliminary comparison to the resident clinic. Lastly, although this data comes from a very large healthcare system that serves approximately 80,000 unique OB patients annually, it represents two clinics in one healthcare system so findings may not be generalizable to different healthcare systems, OB delivery models with those systems, or regions.

## Data Availability

The datasets generated during the study are not currently publicly available but can be made available from the corresponding author upon reasonable request.
